# Supplementary material for: Genotypic and Phenotypic Characterization of Erythromycin-Resistant Staphylococcus aureus Isolated from Bovine Mastitis and Humans in Close Contact
Source: Trop Med Infect Dis. 2022 Dec 29;8(1):26. doi: 10.3390/tropicalmed8010026 (PMC9865518; doi:10.3390/tropicalmed8010026)
Supplement: Supplementary file 1 [file tropicalmed-08-00026-s001.zip › tropicalmed-2106722-supplementary.pdf]

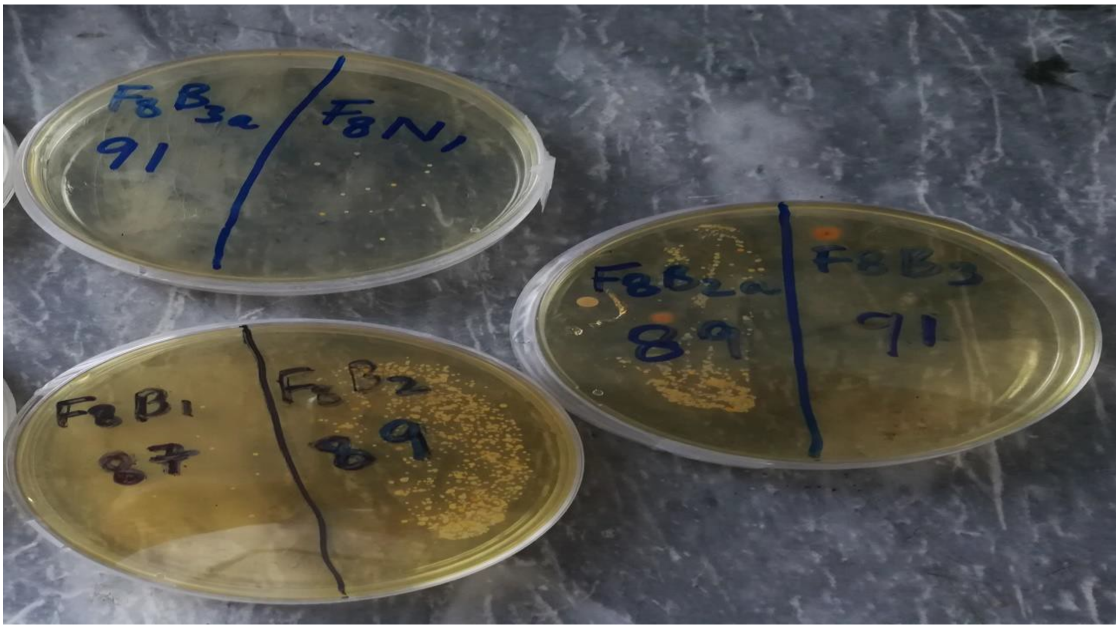

Figure S1. Different colonies of *Staphylococci* on staph 110 agar.

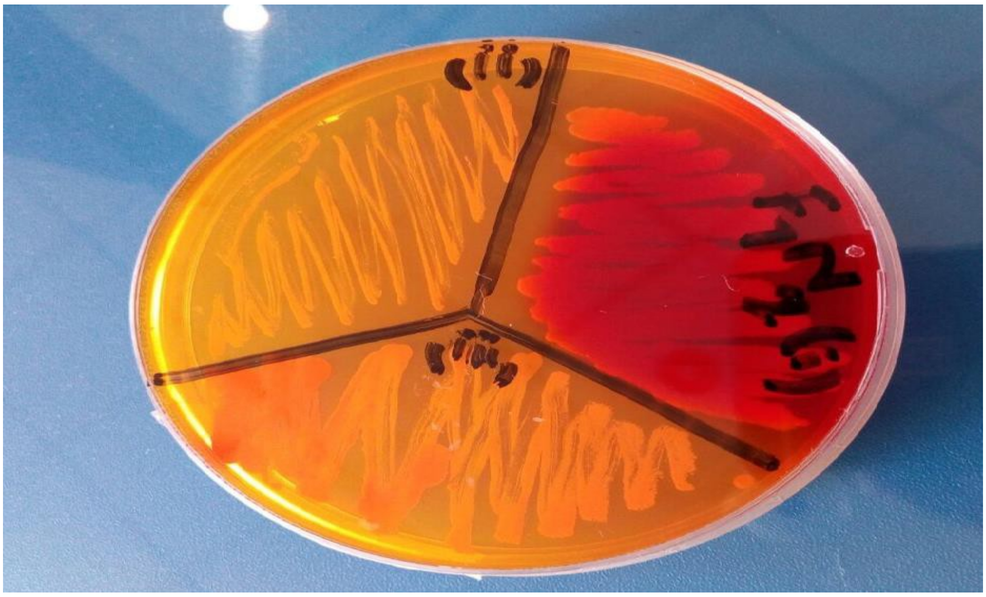

Figure S2. Mannitol test result.

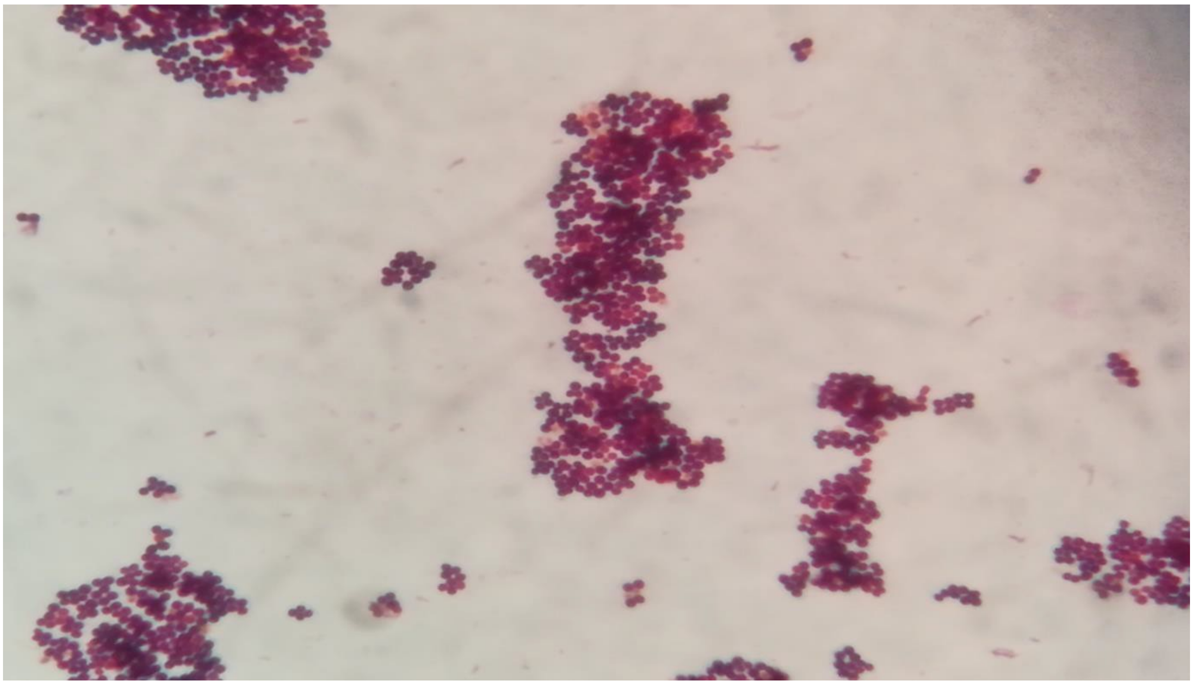

Figure S3. Microscopic view of a cocci shaped Gram-positive *S. aureus.*

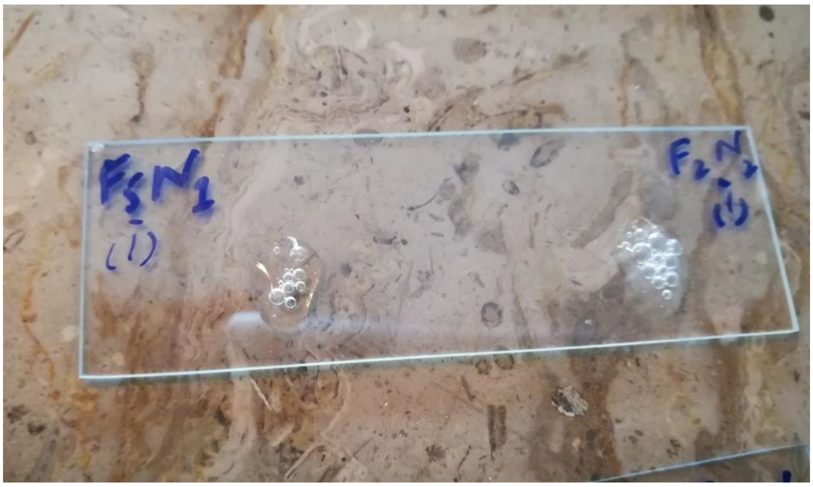

Figure S4. Catalase positive for *S. aureus* isolate.

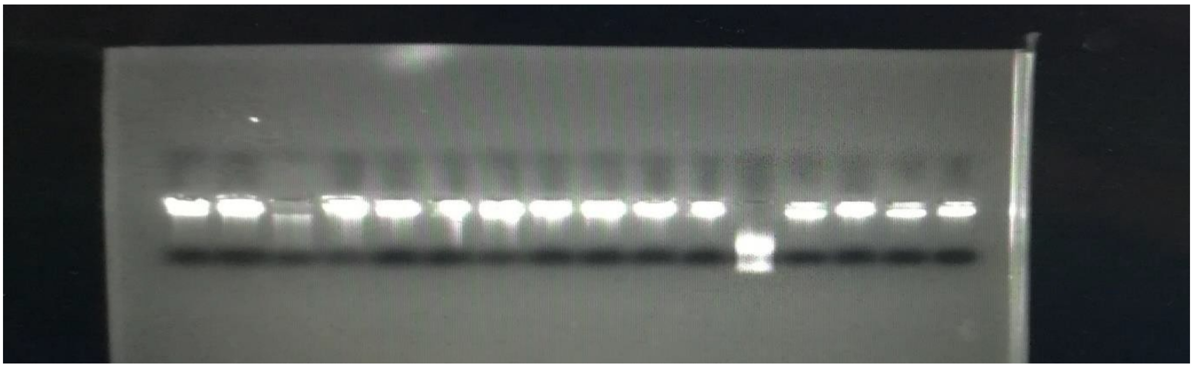

Figure S5. Whole bacterial genomic DNA isolates on 1.5% gel.

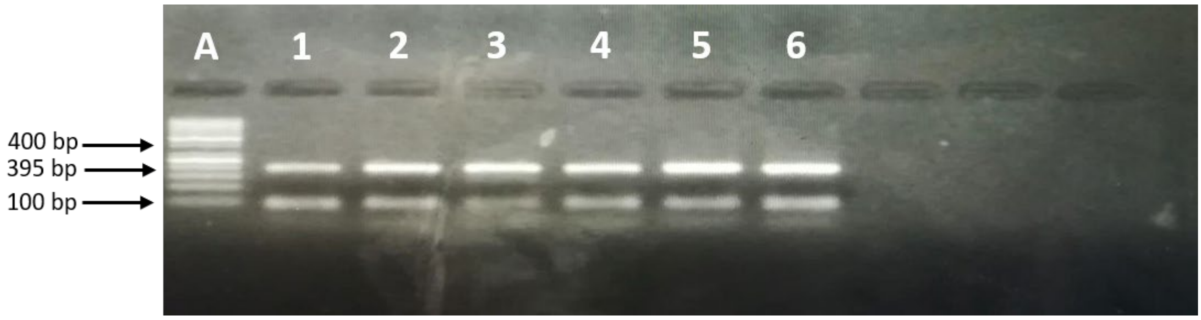

Figure S6. Amplification of *nuc* gene visualized by UV light, showing positive results for *S. aureus* identification. A: 100bp DNA ladder, lane 1-6: 395 bp amplified product of PCR showed milker workers nasal samples and mastitic milk samples positive for thermonuclease genes.

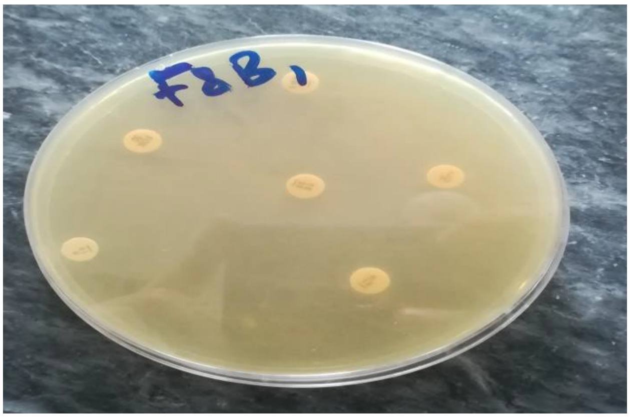

Figure S7. Zones of inhibition of Disk diffusion test. Left plate is showing sensitivity to all antibiotics whileon the right-side plate is resistant to erythromycin and all other antibiotics.

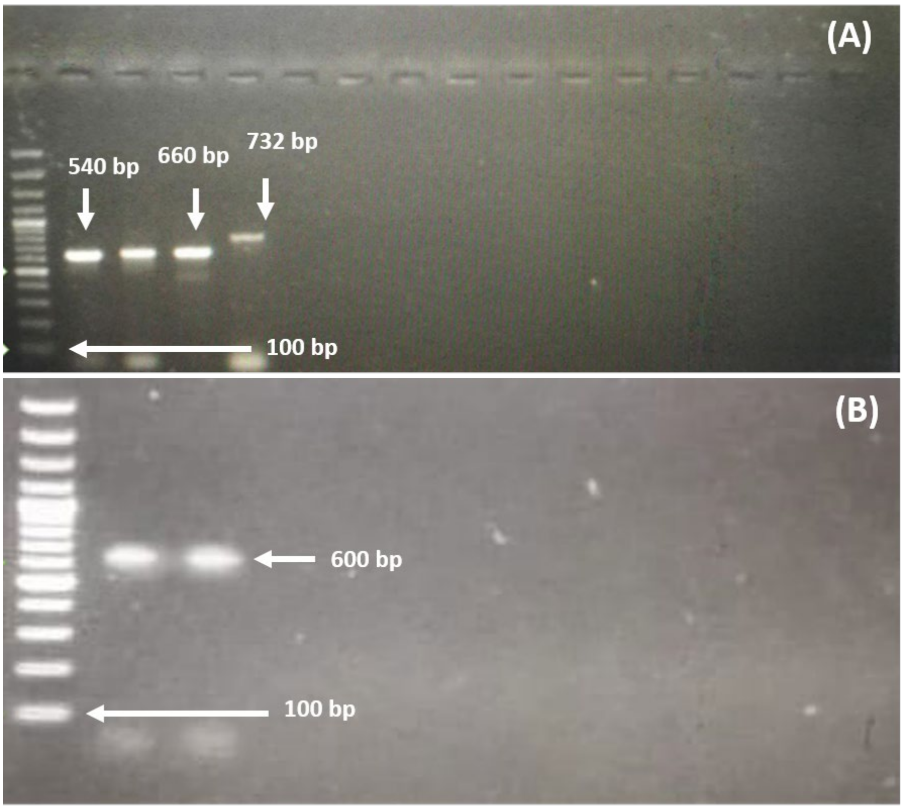

Figure S8. (A): PCR product from 2 sets of primers *ermC* gene and one set of primer of *ermA* gene using 100 bp DNA ladder. **Lane 1 and 2:** 540bp PCR product of *ermC* gene of three samples isolates. **Lane 3:** 660bp PCR product of *ermC* gene of three sample isolates. **Lane 4:** 732 bp PCR product of *ermA* gene of 2 sample isolates. (**B**): PCR products of control from single set of primer *ermB* gene Lane M: 100 bp DNA ladder. Lane 1 & 2: 639 bp PCR product of 2 *S. aureus* isolates

Table S1. Sampling sites and structure of farms.

Sr. No.	Sampling Site	Farm Designation	Herd Size	Buffaloes with MastitisNo.
**1.**	Sheikh Kot, Lahore	F1	20	1
**2.**	Sheikh Kot, Lahore	F2	60	1
**3.**	Sheikh Kot, Lahore	F3	15	1
**4.**	Sheikh Kot, Lahore	F4	6	1
**5.**	Sheikh Kot, Lahore	F5	22	2
**6.**	Sagian bridge, Lahore.	F6	6	1
**7.**	Sagian bridge, Lahore.	F7	40	2
**8.**	UVAS, Lahore.	F8	10	2
**9.**	UVAS, Lahore.	F9	30	3
**10.**	UVAS, Lahore.	F10	5	1
**11.**	Sagian bridge, Lahore.	F11	90	2
**12.**	Harbanspura, Lahore.	F12	5	1
**13.**	Harbanspura, Lahore.	F13	15	12

Table S2 Reaction mixture of PCR for primers of *nuc* gene.

Reagents	Volume (µL)	
DNA 50 ng/µl	2.0	
PCR buffer (2mM)	1.5	
dNTPs (25mM)	1.5	
$MgCl_2$ (20mM)	1.0	
Primers (10 Pmol)	Forward	Reverse
	1.0	1.0
Taq *polymerase* 5 U/µl	0.15	
Distilled Water	7.00	
Total Volume	15	

Table S3. PCR conditions for *nuc* gene.

Steps	Temperature	Time
Initial Denaturation	95°C	5 min
Denaturation	95°C	30 sec
Annealing	55°C	45 sec 30 cycles
Extension	72°C	1 min
Final Extension	72°C	10 min

Table S4. Tm, amplicon sizes and primer sequences.

Sr. No.	Primer Name	5′-3′ Sequence	Tm	Product Size (bp)
1	*ermA-F*	AATGAACCAGTAAGGAGAAGGTT	57.43	
	ermA-R	GCGTCCTCTTGTGAAATTAGAGA	59.91	732
2	*ermB-F*	GAAAAGGTACTCAACCAAATA	57	
	ermB-R	AGTAACGGTACTTAAATTGTTTA C	57	639
3	*ermC-F1*	TCGCATCAGATTGCAGTATAAA	58.47	
	ermC-R1	TAATGCCAATGAGCGTTTTG	59.70	544
4	*ermC-F2*	CGTAACTGCCATTGAAATAGACC	59.91	
	ermC-R2	AAGTGAATGCGCAAAAGACA	59.47	660
5	*Nuc-F*	ATATGTATGGCAATCGTTTCAAT	56	
	Nuc-R	GTAAATGCACTTGCTTCAGGAC	56	395

Table S5. Components of PCR master mix for primers of *ermA, ermB* and *ermC* gene.

PCR Reaction Mixture Reagents	Volume (μL)	
PCR buffer (2mM)	1.5	
$MgCl_2$ (20mM)	1	
DNA 50ng/μl	2.0	
dnTPs (25mM)	1.5	
Taq polymerase 5U/μl	0.15	
Primers (10 pmol)	Forward	Reverse
	1.0	1.0
Distilled Water	7.0	
Total Volume	15	

Table S6. Conditions of PCR for *ermA* gene.

Steps	Temperature	Time
Initial Denaturation	95°C	5min
Denaturation	95°C	30sec
Annealing	53°C	30sec 30 Cycles
Extension	72°C	1min
Final Extension	72°C	10min

Table S7. Conditions of PCR for *ermB* gene.

Steps	Temperature	Time
Initial Denaturation	95°C	5min
Denaturation	95°C	30sec
Annealing	55°C	30sec 30 Cycles
Extension	72°C	1min
Final Extension	72°C	7min

Table S8. Conditions of PCR for *ermC* gene.

Steps	Temperature	Time
Initial Denaturation	95 ^{o}C	5 min
Denaturation	95 ^{o}C	30 sec
Annealing	53 ^{o}C	30 sec 30 cycles
Extension	72 ^{o}C	1 min
Final Extension	72 ^{o}C	7 min
